# Rapid evolution of a large structural polymorphism during a bacterial epidemic

**DOI:** 10.1038/s41437-025-00812-7

**Published:** 2025-11-24

**Authors:** Eric Dexter, Pascal Angst, Peter D. Fields, Fabian Scheuber, Marlon Henseler, Dieter Ebert

**Affiliations:** 1 Department of Environmental Sciences, Zoology, University of Basel, Basel, Switzerland; 2 Dexter Precision Analytics, Basel, Switzerland

**Keywords:** Comparative genomics, Genome evolution, Evolutionary genetics, Structural variation, Structural variation

## Abstract

The field of population genetics is primarily focused on simple genetic variants such as single nucleotide polymorphisms (SNPs), small insertions or deletions (INDELs), and copy-number variants (CNVs). However, large-scale genomic variants are beginning to undergo increased scrutiny as new sequencing methods facilitate their discovery. Here, we report an unusually large and highly variable structural feature in the *Daphnia magna* genome that is strongly associated with immune function. Alternative forms of this large structural polymorphism (LSP) encompass 2–5 Mb regions where homology is undetectable and that contain largely non-overlapping sets of genes. One haplotype (LSP-5-1.1) shows a near-perfect correlation with susceptibility to a common strain of the virulent bacterium, *Pasteuria ramosa*, which is a common and widespread parasite of *D. magna*. Rapid selection against LSP-5-1.1 was observed during a natural *P. ramosa* epidemic, coinciding with a strong population-wide increase in resistance. Despite recurrent episodes of strong selection against *Pasteuria* susceptibility, we observe evidence of balancing selection for this structural polymorphism—suggesting counter selection against the resistant form by a yet unidentified mechanism.

## Introduction

The genetic basis of evolution has predominantly been considered through the lens of relatively simple genetic variants such as single-nucleotide polymorphisms (SNPs), short insertions/deletions (INDELs), copy-number variants (CNVs) and microsatellites (Conrad and Hurles 2007; Morin et al. 2009; Helyar et al. 2011). This approach has provided invaluable insights into the molecular basis of heritable traits, evolutionary dynamics, and the genetic architecture of diseases (Mullaney et al. 2010; Casillas and Barbadilla 2017; Gagliano et al. 2019). However, the understanding of larger-scale differences in genome architecture within species or within populations remains limited (Wold et al. 2021; West et al. 2022; Soto et al. 2023). This is largely due to the dominance of short-read sequencing (Pareek et al. 2011) and reliance on a single reference genomes, which accurately detects small variants but often fails to resolve large structural changes.

Population genomics typically relies on a single reference genome per species, against which small variants are identified (Morrell et al. 2012; Auwera and O’Connor 2020; Uffelmann et al. 2021). However, this approach struggles with read alignment in repetitive or low-complexity regions (Gao et al. 2019). If these repetitive or low-complexity regions are of specific relevance for a given study, this issue can to some extent be mitigated using long-read sequencing technologies. However, most bioinformatic pipelines simply mask low-complexity regions before identifying variants and computing statistics (Tarailo-Graovac and Chen 2009; Bayer et al. 2018; Slotkin 2018).

While long-read sequencing can mitigate alignment issues in repetitive regions, it does not address the broader problem of reference genome bias, where highly divergent haplotypes may fail to align properly (Weisenfeld et al. 2017; Wong et al. 2018; Günther and Nettelblad 2019). Highly divergent haplotypes may be mischaracterized as missing or low coverage data, and beyond a certain threshold, alignment-based variant calling becomes impossible. Thus, standard approaches to calling genetic variants and calculating statistics may be completely unusable for the most highly variable regions of a genome (Bourgeois et al. 2021; Lee et al. 2021). Such highly divergent regions are likely to be of strong interest, and therefore masking genomic regions where reads align poorly may be counterproductive.

Recent advances in long-read sequencing technologies now enabled more flexible analytical approaches that incorporate multiple reference genomes, providing a more comprehensive view of within-species diversity and a framework to deal with the persistent issue of high haplotype divergence (Monnahan et al. 2020; Chen et al. 2021; Kaye and Wasserman 2021). This shift in capability brings into focus large-scale elements of genomic variation, such as supergenes, which are particularly difficult to parse using traditional approaches to variant calling (Gutiérrez-Valencia et al. 2021).

Supergenes are physically linked loci that interact to produce discrete phenotypes and are typically maintained through balancing selection (Schwander et al. 2014; Thompson and Jiggins 2014; Gutiérrez-Valencia et al. 2021; Berdan et al. 2022). Their preservation is particularly relevant when multiple traits must change in coordination, such as in behavioral or morphological shifts (Thompson and Jiggins 2014). Well known examples include a supergene in the fire ant *Solenopsis invicta* that controls colony social organization (Ross and Keller 1998; Kay et al. 2022), wing mimicry in butterflies (*Heliconius numata*) (Joron et al. 2011), mating and plumage polymorphisms in birds (*Zonotrichia albicollis*) (Maney et al. 2020), and flower morphs of plants such as *Primula vulgaris* (Li et al. 2016).

While the supergene model is fascinating and conceptually useful, not all large genomic variants fit within this framework. For example, in *C. elegans* large-scale structural variants are observed, so called “hyper-divergent haplotypes” (HDHs). These regions span over 1 Mb, exhibit high sequence diversity, and show little to no recombination between haplotypes (Lee et al. 2021). Unlike supergenes, which typically involve a small number of haplotypes (2–3) associated with distinct phenotypes, these HDH regions host many unique haplotypes comprised of divergent sequence content rather than structural rearrangements such as inversions or translocations (Lee et al. 2021; Stevens et al. 2023).

An example of extreme haplotype divergence is found in the planktonic crustacean *Daphnia magna*, which often experiences high rates of seasonal infection by the highly pathogenic bacteria *Pasteuria ramosa* (Ebert 2005). Resistance to *P. ramosa* is controlled by at least six interacting loci (Bento et al. 2017; Bento et al. 2017; Ameline et al. 2021; Fredericksen et al. 2023), four of which are located the *Pasteuria* resistance complex, a region of major structural polymorphism. This complex shows extreme divergence between haplotypes, including long stretches where homology could not be detected, the apparent absence of recombination, and the long-term maintenance of variation through balancing selection (Bento et al. 2017; Bourgeois et al. 2021; Naser-Khdour et al. 2024).

Long-term monitoring of a *D. magna* population where regular seasonal outbreaks of *P. ramosa* occur (Lake Aegelsee, Switzerland) has shown rapid and repeatable shifts in resistance phenotypes across the duration of each outbreak (Ameline et al. 2021; Ameline et al. 2022). During each epidemic, this population consistently shifts towards resistance to the *P. ramosa* isolate P20, which is native and abundant in this lake (Fredericksen et al. 2021; Ameline et al. 2022). This phenotypic shift appears to reflect changes in underlying allele frequencies among clone lines, as *Daphnia* do not exhibit adaptive immunity (Ebert 2005). Laboratory experiments indicate that these cyclical changes can be explained as an epistatic interaction between the *Pasteuria*-resistance complex on chromosome 4 and a separate locus on chromosome 5 (Ameline et al. 2021). While the genetic model of this chromosome 5 locus (the E locus) is well resolved, its precise location remains elusive, with candidate positions scattered across the chromosome (Ameline et al. 2021). This suggests that the E locus may reside within a difficult to map structural polymorphism on chromosome 5.

To investigate this possibility, we conducted a comparative analysis of 21 independently assembled *Daphnia* genomes developed with long-read sequencing. Blocks of homologous genes were identified across all genomes and used to construct a syntenic map of chromosome 5. We then genotyped 258 previously sequenced Lake Aegelsee *D. magna* genotypes and compared alternative forms of chromosome 5 against phenotypic resistance data. Our analysis revealed the presence of an unusually large structural feature in the *D. magna* genome, which exhibits haplotypes sufficiently divergent to prevent mapping of reads (even long reads) across a large portion of the chromosome relative to any single reference. One of these haplotypes shows a near-perfect correlation with susceptibility to *P. ramosa* isolate P20, and likely contains the physical location of the E locus. Despite nearly 15 years of intensive research into the genome of *D. magna* (Routtu et al. 2010; Fields et al. 2015; Dukić et al. 2016; Orsini et al. 2016; Chaturvedi et al. 2023; Dexter et al. 2023), this chromosome-scale genomic feature remained undetected until the development of a diverse panel of reference genome assemblies.

## Methods

### Study system

*Daphnia magna* is a common planktonic crustacean that inhabits standing fresh and brackish waters across the northern hemisphere. Wild populations of *D. magna* often experience high rates of seasonal infection by the endospore-forming bacteria *Pasteuria ramosa*, which castrate and eventually kill their host (Ebert et al. 2016) infection begins when *Daphnia* ingest planktonic spores of *P. ramosa* during filter feeding, which enter the digestive tract along with food and other particulate matter and attach to the gut lining. Attached *P. ramosa* cells then emerge from an outer spore capsule and rapidly proliferate in the body cavity of the host. *Pasteuria* infection results in rapid sterilization of the host followed by death in 30–60 days, with an apparent mortality rate of 100% (Ebert et al. 1996). *Pasteuria* infection spreads exclusively through horizontal transmission from host to host, and has been observed over most of the *D. magna* geographic range (Andras et al. 2018; Fredericksen et al. 2021). The *D. magna* genotypes ( = clones) used in the current study have been used in various earlier studies (Fredericksen et al. 2021; Fredericksen et al. 2021; Ameline et al. 2022).

*Pasteuria* infection follows a matching-allele model, such that *D. magna* clone lines are either completely susceptible or completely resistant to particular strains of *P. ramosa* (Luijckx et al. 2013; Bento et al. 2017; Bento et al. 2020). This binary outcome likely arises from the attachment step of the infection process, in which collagen-like proteins on the *Pasteuria* spore capsule interact with highly polymorphic extracellular proteins in the host gut (Andras et al. 2020). Laboratory-based attachment tests using a wide range of host and parasite genotypes support this model of infection, showing that infection strongly correlates with attachment, and that infection is only possible between specific combination of host and parasite genotypes (Duneau et al. 2011; Fredericksen et al. 2021). The *P. ramosa* isolates used here have been used earlier in studies of the Lake Aegelsee population (Ameline et al. 2021; Fredericksen et al. 2021; Ameline et al. 2022).

The *D. magna* time-series samples were collected from Lake Aegelsee (47.558048, 8.861063) via horizontal net tows from a fixed location across the growing season of each year (Mar – Sep) as part of an ongoing monitoring program (Ameline et al. 2022; Dexter et al. 2023). *P. ramosa* infection status was determined for each collected individual via light microscopy, and parasite-free clone lines were established for the purpose of resistance testing by first eliminating any pre-existing infections with tetracycline antibiotic treatments (Duneau et al. 2011; Bento et al. 2020). *Pasteuria*-resistance phenotypes were then assessed for each of these *D. magna* clone lines through gut attachment tests with fluorescently labelled *Pasteuria* spores using a standardized panel of five isolates: C1, C19, P15, P20, and P21 (Duneau et al. 2011; Bento et al. 2017; Bento et al. 2020; Fredericksen et al. 2023).

### Gene prediction and functional annotation

We performed gene prediction and functional annotation of 21 previously-assembled long-read *D. magna* genomes and one *D. similis* outgroup. Eleven of the *D. magna* genomes originated from the Lake Aegelsee study population, while the remaining were drawn from populations across the northern hemisphere (Table S2). Highly repetitive content was masked from each genome assembly prior to gene prediction using RepeatModeler version 2.0.2 and RepeatMasker version 4.1.4 (Tarailo-Graovac and Chen 2009; Flynn et al. 2020). Gene prediction was performed using Augustus version 3.4.0 (Hoff and Stanke 2019) with a pre-existing Daphnia magna species model (Dmagna_iso), which had been previously parameterized using curated gene structures from the FI-Xinb3 clone. Predictions were integrated with SNAP and GeneMark outputs within the Maker pipeline to produce the final gene predictions (Hoff and Stanke 2019). We then conducted a functional annotation of the predicted genes using InterProScan version 5 operating under default parameters (Jones et al. 2014). The Genespace (v 1.2.3) package for R (v 4.1.2) was then used to visualize genome synteny and orthology across the complete set of functionally annotated genomes (Lovell et al. 2022). Genespace analysis was conducted with settings designed to maximize resolution of synteny blocks across a single chromosome: blkSize = 2, blkRadius = 50. onewayBlast = TRUE. Synteny blocks were then visualized using the plot_riparian() function in Genespace with custom graphical parameters.

### Comparison of reference genome assemblies

For each of the three LSP haplotypes that were observed in the Lake Aegelsee population, one genome was selected to serve as the canonical reference assembly. These canonical references were selected based on contiguity and completeness, with each containing most of the right arm of chromosome 5 (including the LSP region) in a single contig. Scaffolding was accomplished by mapping individual contigs against a highly-curated chromosome-level *D. magna* genome assembly that incorporates optical mapping and recombination map data (NCBI accession: LRGB00000000.1). Sequence homology between variant chromosome 5 assemblies was then visualized as a matrix of pairwise synteny dot plots. Contig mapping and dot plot visualization were performed using Minimap version 2 (Li 2018) and D-Genies (Cabanettes and Klopp 2018) with default parameters (minimum k-mer >= 15 and chaining score >= 40).

### Gene content and orthogroup analysis

To assess gene content differences among the three LSP haplotypes, predicted protein-coding genes from each canonical reference genome were clustered into orthogroups using OrthoFinder v2.5.5 (Emms and Kelly 2019). Genes were classified as shared if their orthogroup contained genes from two or more haplotypes, and as unique if their orthogroup contained genes from only a single haplotype. Private genes were quantified for each haplotype to distinguish true presence/absence variation from potential gene duplication or loss (i.e., one-to-many relationships). Presence/absence patterns were visualized using Venn diagrams generated in R with the ggVennDiagram package (Gao et al. 2024). As a control, the same analysis was applied to an equivalently sized region on the left arm of chromosome 5, confirming that shared genes are readily detected when present.

### Reference genome re-mapping

Whole genome short-reads from 258 previously sequenced wild-collected *D. magna* were mapped against each of the reference assemblies. These samples were originally collected from Lake Aegelsee, Switzerland (WGS84: 47.558048, 8.861063) during a seasonal epidemic of *P. ramosa* infection and sequenced across multiple runs of an Illumina Novaseq 6000 to generate paired-end 100 bp reads (Dexter et al. 2023). After de-multiplexing the sequence-read pairs, we used Trimmomatic ver. 0.39 (Bolger et al. 2014) to remove low-quality reads and adaptor sequences. Duplicate reads were then removed using Picard ver. 2.9.2. The trimmed reads were mapped against each of the three reference *D. magna* assemblies using BWA-MEM version 0.7.15 with default parameters (Li 2013). Mapping depth was then calculated for each sample across chromosome 5 using SAMtools ver. 1.10 with minimum mapping quality (MQ) set to 40. (Li et al. 2009). Genotypes at the LSP region were assigned to each sample based on the ratio of read depth within the LSP to the read depth in the adjacent flanking regions on the same chromosome.

### Variant calling and LD calculation

We called genomic variants (SNPs, indels, and mixed-type variants) relative to each reference genome using the GATK haplotype caller ver. 4.4.0 following the best practices pipeline for diploid samples (Auwera and O’Connor 2020). The resulting VCF files were then filtered to remove low-quality bases, poor alignment scores, or extreme strand biases. The remaining variants were then thinned with VCFtools ver. 0.1.16 (Danecek et al. 2011) prior to LD calculation using the following parameters: --thin 2000 --min-r2 0.0 --maf 0.05 --max-missing 0.5 --minGQ 30. We then calculated linkage disequilibrium (LD) across the length of chromosome 5 using VCFtools with the following settings: --thin 2000 --min-r2 0.0 --maf 0.05 --max-missing 0.5 --minGQ 30. Pairwise LD values were visualized as a heatmap plot using the ggplot2 ver. 3.3.5 package in R ver. 4.1.2 (R Core Team 2016; Wickham et al. 2018).

### Assessment of resistance phenotypes

We collected *Pasteuria* resistance phenotypes (aka. “resistotype”) data for a random subset of the sequenced *D. magna* clone lines, with each clone tested in triplicate. Resistotypes were assessed through attachment tests with fluorescently labelled *P. ramosa* spores using a standardized panel of five laboratory-cultured isolates: C1, C19, P15, P20, and P21 (Duneau et al. 2011; Ameline et al. 2022). Isolates can be used to infer allelic states at the known pasteuria resistance loci. For example, the allelic state at the A, B, and C loci can be diagnosed relative to *Pasteuria* isolates C1 and C19 (Bento et al. 2017), while the D and F loci can be diagnosed relative to isolate P15 and P21 (Bento et al. 2020; Fredericksen et al. 2023). The E locus can be diagnosed relative to isolate P20, but due to a masking interaction with the C locus, only in genotypes which are homozygotes or heterozygotes for the dominant C allele (i.e. resistant to isolates C1 and C19) (Ameline et al. 2022).

### Use of AI

ChatGPT version 4.0 (OpenAI) was used to provide proofreading and grammar suggestions to the manuscript text. No AI tools were used in the generation of figures, citations, code, or analysis.

## Results

### Observation of extreme linkage disequilibrium on chromosome 5

**Fig. 1:**
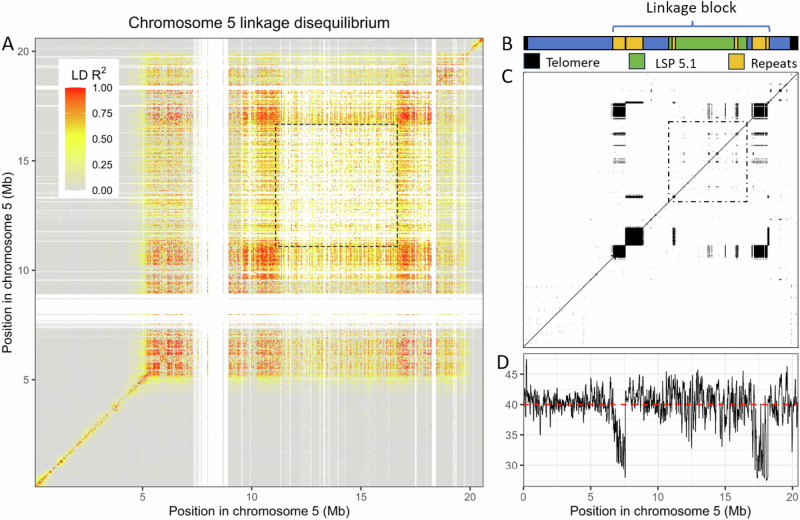
Linkage disequilibrium (LD) plotted across chromosome 5 of the D. magna genome. **A** Linkage disequilibrium (LD) plotted across chromosome 5 of the *D. magna* genome. A large linkage block (yellow to red) is visible across most of the right arm of the chromosome, which extends across the centromere to include a portion of the left arm. White areas indicate regions of the chromosome where statistics cannot be computed due to poor read mapping, such as in the predicted position of the centromere around 7–8 Mb. Poor read mapping also characterizes this large structural polymorphism (LSP) region, which is outlined by the dashed box. **B** Schematic diagram of chromosome 5 showing the location of the LSP region (in green) within the LD block. **C** Dotplot self-alignment of chromosome 5 showing large blocks of highly repetitive genetic elements (black squares) that flank both edges of the linkage block. The LSP region is outlined by the dashed box. **D** GC % is plotted across chromosome 5 with the genome-wide mean shown in red. An approximately 10% decrease in GC content is associated with the highly repetitive elements that flank the linkage block. All positions shown are in terms of physical distance along the chromosome in relation to the reference assembly CH-H-2014-t2-17-3-4i-13, a genotype susceptible to *P. ramosa* P20.

Because previous studies had mapped the E locus of *Pasteuria* resistance to a difficult-to-localize position on chromosome 5, we searched for structural variation on chromosome 5 by scanning for blocks of linkage disequilibrium (LD) across 258 *D. magna* genomes sampled from one population (Lake Aegelsee). This scan revealed a previously undetected linkage block spanning across a large part of the chromosome (Fig. 1A). This linkage block spans nearly the entire right chromosome arm, except for a small segment near the telomere, and a portion of the left arm near the centromere. This LD block is flanked on both sides by approximately 1 Mb stretches of highly repetitive sequence content (Fig. 1B, C). Allelic variants within this LD block exhibit high linkage (R^2^ > 0.9) across distances up to 13 Mb. This linkage block appears to be sharply defined. For comparison, variants located elsewhere on the chromosome (and on other chromosomes) typically show independent segregation at distances of less than 100 Kb. Given that this population reproduces sexually at least once per year and is estimated to be about 60 years old, this linkage pattern suggests a mechanism locally suppressing recombination across a large portion of the chromosome.

### Multiple haplotypes in Lake Aegelsee

**Fig. 2:**
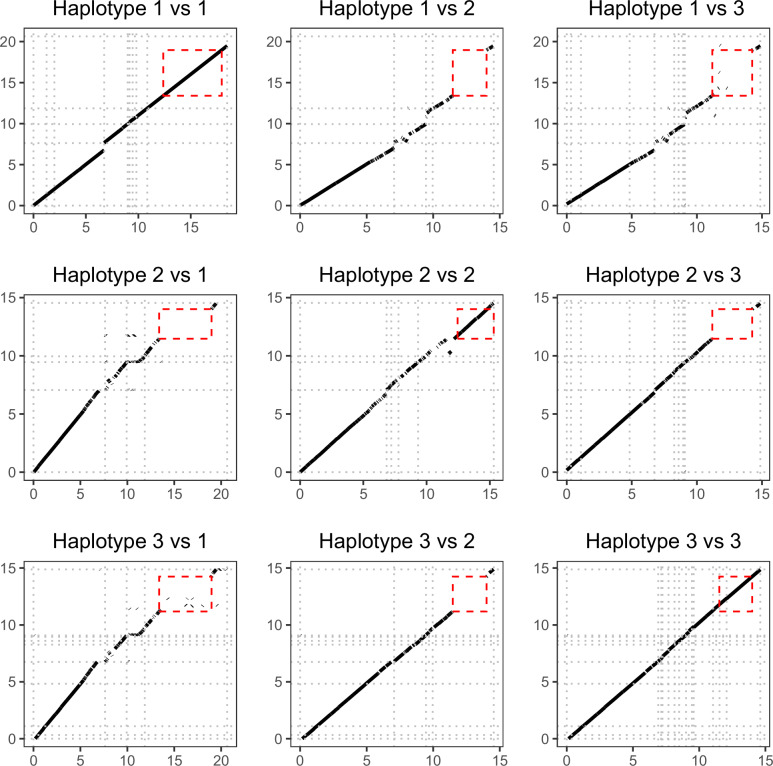
Dotplot matrix of chromosome 5 DNA sequence synteny from genome assemblies containing the three large structural polymorphism (LSP) found in the Lake Aegelsee population. The borders of individual contigs within each assembly are shown with light grey stippled lines. Alignments on the diagonal axis (1 vs 1, 2 vs 2 and 3 vs 3) represent independent genome assemblies from two different individuals that share the same haplotype at the LSP region. All genome assemblies show strong homology across the left arm of chromosome 5, while assemblies with differing haplotypes show a major break in synteny across much of the right chromosome arm. The LSP region (highlighted in red) was assembled as a single contig in almost all cases, thus precluding scaffolding errors as an artificial source of variation.

We discovered a large region within the chromosome 5 LD block where short-read mapping rates were highly variable between samples. This region varies from 2 to 5 Mb in size, depending on the haplotype, with boundaries defined by conserved flanking sequences shared across all examined genomes. For some individuals, mapping coverage within this region corresponded to the genome-wide average, while in other individuals mapping coverage was almost zero. These results suggested that some of the haplotypes segregating in Lake Aegelsee were sufficiently divergent to preclude short-read mapping relative to a single reference genome. This hypothesis was confirmed by pairwise alignment of a panel of reference genomes assembled from Lake Aegelsee, which revealed the presence of a large structural polymorphism (LSP) contained within the chromosome 5 LD block (Fig. 2).

As standard approaches for genotyping were not possible within this region, we iteratively mapped whole-genome Illumina short-reads from the 258 *D. magna* samples against three different genome assemblies, each homozygous for a different haplotype at this LSP region. Genotypes for the 258 samples were determined based on relative mapping depths, a useful approach when extreme divergence prevents read mapping and variant calling (Li et al. 2016; Cocker et al. 2018; Gutiérrez-Valencia et al. 2021). With this approach we could unambiguously genotype the highly divergent region for 256 of 258 samples (Fig. S1), with two samples excluded due to poor sequencing yield.

**Fig. 3:**
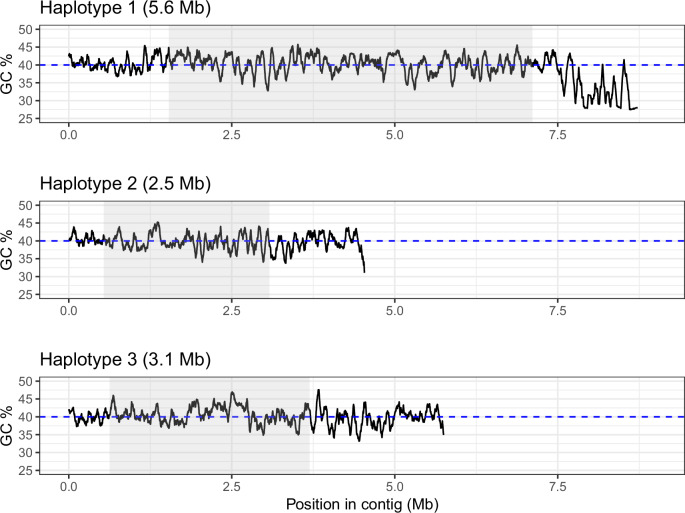
GC % plots showing for each of the LSP haplotype reference assemblies the entire contig which contains the LSP region (shaded in grey). The LSP region is defined as the area between two 1-Kb flanking sequences which are conserved across all three genomes and which occur nowhere else in the *D. magna* genome. The length of the LSP is given in the titles of each sub-panel in brackets. The dashed blue line indicates the genome-wide mean GC %. While the overall contig length varies between genome assemblies, in each case the LSP region is clearly captured in its entirety.

Our depth-based genotyping results confirmed that only three haplotypes segregate in appreciable frequencies in Lake Aegelsee at this LSP region, with all samples being clearly categorized as being heterozygous or homozygous for various combinations of the three haplotypes (Fig. S1). All combinations of these haplotypes appear to be viable, with each possible combination observed in at least 10 samples (Table S1). These haplotypes are designated under a hierarchical naming scheme as LSP-5-1.1, LSP-5-1.2, and LSP-5-1.3, indicating they are the first three haplotypes of the first large structural polymorphism discovered on chromosome 5. For ease of notation, we will use the following shorthand names throughout the manuscript: Haplotype 1 for LSP-5-1.1, Haplotype 2 for LSP-5-1.2, and Haplotype 3 for LSP-5-1.3. These three haplotypes contain 2.5–95.6 Mb of unique sequence content, with Haplotype 1 being approximately twice the size of the other two haplotypes (Fig. 3). Each haplotype was observed in multiple genome assemblies, so we selected one *D. magna* genome from Lake Aegelsee as the canonical reference for each. These three genomes were selected based on contiguity and completeness, with each genome assembly containing most of the chromosome 5 left arm in one contig, and the right arm in a second contig.

### Linking the LSP region to bacterial parasite resistance

Clones of *D. magna* differ widely in their resistance to different isolates of the bacterial pathogen *Pasteuria ramosa*. One of the resistance loci, the E locus, was previously determined to occur on chromosome 5 at an ambiguously defined location (Ameline et al. 2021; Ameline et al. 2022). As the allelic state at the E locus is defined by susceptibility (dominant) or resistance (recessive) to *P. ramosa* isolate P20, we examined the correlation between haplotypes at the chromosome 5 LSP region and resistance to P20, which is common in Lake Aegelsee (Ameline et al. 2021). Our results show that Haplotype 1 is strongly associated with susceptibility to P20 infection, and follows a dominance pattern that matches the previously described genetic model for the E locus (Ameline et al. 2021). We found that 16 of the 20 clones (80%) carrying at least one copy of Haplotype 1 were completely susceptible to infection by *P. ramosa* isolate P20. In contrast, 68 out of 69 clones (99%) containing combinations of Haplotypes 2 and 3 (i.e. genotypes 2/2, 3/3, and 2/3) were completely resistant to infection by isolate P20 (Table 1).

**Table 1:** Correspondence between the observed genotype at the chromosome 5 LSP region, the predicted E locus state, and the measured P20 resistance phenotype.

Genotype (observed)	P20 Resistant	P20 Susceptible	E locus (hypothetical)
Haplotype 2 homozygote	12	0	ee
Haplotype 3 homozygote	16	0	ee
Haplotype 2 / Haplotype 3	36	1	ee
Haplotype 1 homozygote	0	6	EE
Haplotype 1 / Haplotype 2	2	6	Ee
Haplotype 1 / Haplotype 3	2	4	Ee

Cells shaded in blue represent the expected phenotypes based on the previously-developed E locus genetic model for resistance to isolate P20. The E locus model is a two-allele system with the dominant E allele conferring susceptibility to isolate P20. At the genetic level, this appears to maps onto three haplotypes, with Haplotype 1 corresponding to the dominant E allele. Note that P20 susceptibility is completely masked by an interaction with the recessive allele at the C locus; therefore, only the 85 clones which contain at least one copy of the dominant *C* allele are shown here. 5 of these 85 clones (6%) do not match the E locus genetic model, which might be attributed to measurement error or the epistatic influence of another unidentified locus.

These results strongly indicate that the previously described E locus for *Pasteuria* resistance is contained within the LSP region of chromosome 5, with Haplotype 1 corresponding to the dominant *E* allele for P20 susceptibility. Haplotype 2 and Haplotype 3 both appear to correspond with the recessive *e* allele (P20 resistant). Previous studies showed that the E locus is epistatically masked by the recessive *c* allele at the C locus positioned on chromosome 4 (Ameline et al. 2021). Genotypes which are *cc* are always susceptible for P20, irrespective of the genotype at the E locus. This means that phenotypic information cannot inform about the haplotypes at the E locus when the genotype *cc* is present at the C locus. Thus, Table 1 includes only clones where the E locus phenotype is observable (i.e., CC or Cc at the C locus). The non-perfect association of P20 resistance with the three variants of the LSP region (Table 1) remains unexplained, but may have multiple reasons, including: incorrect typing of resistance, unknown epistatic effects with other loci (e.g. the B locus, Ameline et al. 2021), or that the E locus is not within the LSP, but very close to it.

### Rapidly shifting LSP haplotype frequencies in Lake Aegelsee

**Fig. 4:**
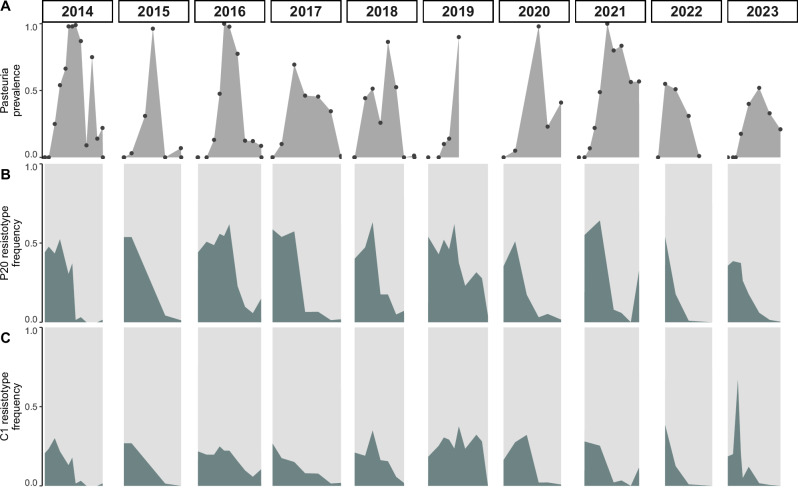
Longitudinal time series of *P. ramosa* field data from Lake Aegelsee. Data from 2014–2018 were previously published (Ameline et al. 2022) and are redrawn here for completeness. **A** Prevalence of *P. ramosa* infection among random samples of approximately 100 adult female *D. magna*. Infection prevalence peaks mid-summer, often nearing 100%, before declining sharply each autumn. Black points indicate individual sampling events. The *P. ramosa* prevalence data for 2019 represent only a partial summer season. **B** Seasonal fluctuations in resistance to the *P. ramosa* isolate P20. Each summer, strong selection reduces the frequency of P20-susceptible hosts, but susceptibility re-emerges at similar levels each spring. Resistance assays were conducted using a separate sample of ~100 adult females, distinct from those used for prevalence estimates in (**A**). The dark shading represents the frequencies of hosts genotypes susceptible to *Pasteuria* isolate P20, i.e. host genotypes with at least one copy of Haplotype 1. **C** Seasonal fluctuations in resistance to the *P. ramosa* isolate C1. The pattern mirrors that of P20 resistance but with a weaker magnitude. The dark shading represents host genotype frequencies susceptible to *Pasteuria* isolate C1, and represents homozygotes for the recessive *c* allele. These resistotypes are also susceptible to P20 because of epistatic interactions between the C and the E locus. Resistance assays were conducted on the same 100 *D. magna* individuals used for P20 resistance testing in (**B**).

A long-term series of field observations from Lake Aegelsee provides direct phenotypic evidence of balancing selection in response to seasonal *Pasteuria ramosa* outbreaks. Each year, the frequency of P20 *Pasteuria*-resistant genotypes sharply increases as the epidemic emerges in early summer (Fig. 4). This phenotypic shift is mirrored at the genomic level by a dramatic change in haplotype frequencies within the LSP region of chromosome 5. At the beginning of each field season (1–2 months prior to the *P. ramosa* epidemic), all three haplotypes are observed in approximately equal frequencies (Table 2). However, this balance shifts drastically during the epidemic, which reaches a peak infection prevalence of nearly 90% (Table S2). During this period of strong selection, the frequency of Haplotype 1, which is strongly associated with P20 susceptibility, declines rapidly. Of the 382 haplotypes sampled after the mid-summer peak of the epidemic, Haplotype 1 was found only 6 times (1.6%), while Haplotype 2 and Haplotype 3 had increased to 50.8% and 47.6%, respectively. This strong seasonal selection against Haplotype 1 is counterbalanced by its recurrent reappearance each spring, suggesting an underlying mechanism of balancing selection that maintains haplotype diversity in the population.

**Table 2:** Changes in haplotype frequency at the LSP region measured before and after a seasonal outbreak of *P. ramosa* at Lake Aegelsee, shown as counts and percentages.

Haplotype	Pre-epidemic	Post-epidemic
Haplotype 1	46 (35.8%)	6 (1.6%)
Haplotype 2	37 (28.5%)	194 (50.8%)
Haplotype 3	47 (36.1%)	182 (47.6%)
Total	130 (100%)	382 (100%)

Estimates of infection prevalence at the peak of the epidemic ranged from 90 to 98%, with laboratory-based estimates of mortality due to *P. ramosa* infection near 100%. A chi-square test supports significant differences between periods (χ² = 122.75, df = 2, *p* < 0.001). *D. magna* are diploid and therefore two haplotypes were recorded for each of 256 collected individuals.

### Candidate genes for the E locus

**Fig. 5:**
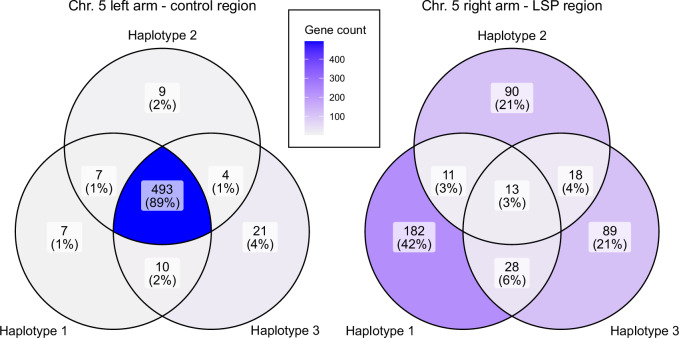
Venn diagram of shared gene content between three genome assemblies representing the three haplotypes in Lake Aegelsee across the LSP region of the right chromosome 5 arm (right diagram). An equivalently sized region from the left arm of chromosome 5 was used as a control region (left diagram). 551 genes were identified from the 4 Mb control region of the left arm, with 493 genes (89%) found in all three genome assemblies. In contrast, 431 genes were identified from the 2–5 Mb LSP region of the right arm, of which only 13 (3%) were found in all assemblies.

After determining that the E locus for *Pasteuria* resistance likely resides within the LSP region of chromosome 5, we sought to identify candidate genes pertaining to *Pasteuria* resistance, focusing on differences separating Haplotype 1 (P20 susceptible) from Haplotype 2 and Haplotype 3 (P20 resistant). Through a combination of gene prediction and functional annotation tools, we identified a total of 431 genes among the three haplotypes, of which only 13 (3%) were shared among all three (Fig. 5). Our gene prediction models show that Haplotype 1 contains 182 private genes, while Haplotype 2 and Haplotype 3 contain 90 and 89 private genes, respectively. From this relatively large list of candidate genes, two Fucosyltransferase genes which are found only on Haplotype 1 are of particular interest (Table S3). Fucosyltransferases, which are involved in the glycosylation of proteins, have been generally linked to the attachment of bacteria to host tissues (Ruiz-Palacios et al. 2003; Maroni et al. 2015) and specifically linked to the attachment of *P. ramosa* to *D. magna* (Bento et al. 2017; Fredericksen et al. 2023; Naser-Khdour et al. 2024).

As a comparison, we repeated this analysis using an equivalently sized 4 Mb control region from the left arm of chromosome 5, which was outside the LD block, and contained within a single contig in each of the three reference genomes. We identified a total of 551 genes in this region, of which 493 (89%) were shared among all three genome assemblies (Fig. 5). These results confirm that the LSP region of chromosome 5 is a highly unique structural feature of the *D. magna* genome. Given the large number of differing genes, the full phenotypic effects of these haplotypes likely extend beyond *P. ramosa* resistance and remain unknown.

### Many divergent haplotypes are found within the chromosome 5 LD block

**Fig. 6:**
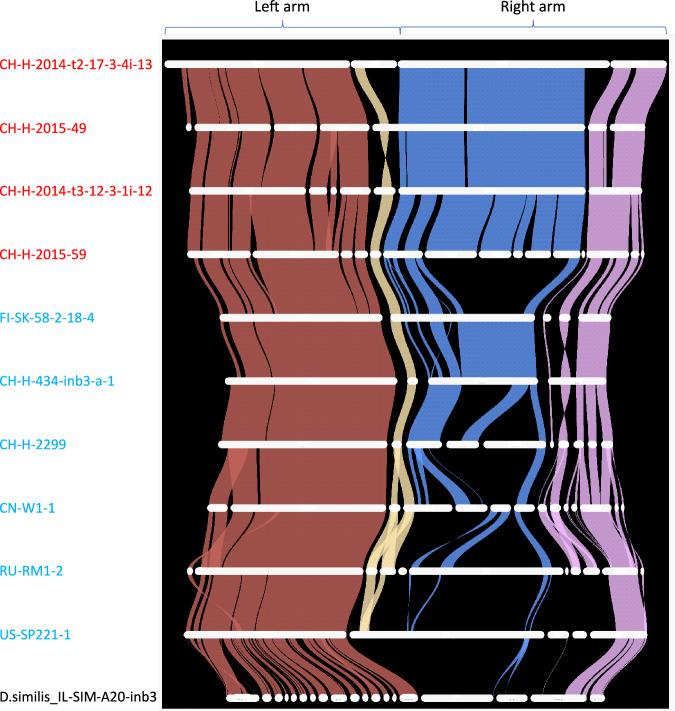
Syntenic map of chromosome 5 across ten *D. magna* genome assemblies and one *D. similis* outgroup. Each strand indicates a syntenic block containing multiple homologous genes, with strand colour relating to the four contigs shown on the uppermost genome. For clarity, only the 10 most contiguous assemblies of chromosome 5 of the 21 *D. magna* genomes (plus one *D. similis* outgroup) are shown. Contigs less than 50 kb in length are not shown. Genome names shown in red contain Haplotype 1 and are susceptible to infection by *P. ramosa* isolate P20. Genome names shown in blue contain various other haplotypes which are all resistant to infection by isolate P20. The first two letters of the *D. magna* clone name indicate the country of origin. Clones starting with CH-H are from the Lake Aegelsee population in Switzerland. Note the strong homology across all genomes on most of the left chromosome arm (in dark red), and the extreme structural variation on the right chromosome arm between different haplotypes.

To explore the wider context of this highly-divergent haplotype region, we conducted a comparative structural analysis of chromosome 5 from 21 *Daphnia* genome assemblies from populations across the Northern Hemisphere. In total, we recovered 10 highly divergent haplotypes from the 21 genome assemblies, each showing a large degree of divergence from all others at the LSP region (Fig. 6). Some of these haplotypes appear to be widely distributed across a broad geographic range, with several cases in which the same haplotype was recovered from multiple continents (Table S2).

Our results showed a remarkable degree of structural divergence across most of the right chromosome arm, with little to no discernible homology between most haplotypes (Fig. 6). In contrast, the left arm of the same chromosome showed strong homology across all genome assemblies, including one *D. similis* outgroup. We used OrthoFinder (Emms and Kelly 2019) to quantify this divergence by calculating the number genes that were conserved across different reference assemblies of chromosome 5. Conserved genes were defined as homologous genes which could be found on chromosome 5 of our primary reference genome (CH-H-2014-t2-17-3-4i-13) and at least 50% of the genomes in our panel. We found that 76% of genes located on the left arm (880 of 1164) were conserved across the panel of genome assemblies, compared to 29% of genes located on the right arm (174 of 602). All *D. magna* genomes showed equally poor synteny with the *D. similis* genome across the LSP region. Of the 231 predicted genes within the LSP region of the *D. similis* genome, each *D. magna* genome contained only 8–16 homologous genes.

## Discussion

Regions of high structural divergence have been identified in an increasing number of species, yet few have been characterized in detail. This limited understanding constrains our ability to study their evolution and persistence, which is believed to be strongly driven by balancing selection. We discovered a large structural polymorphism (LSP) on chromosome 5 of the *D. magna* genome characterized by broad haplotype divergence, with apparently no recombination. This region appears to contain the previously described E locus for resistance to the highly virulent parasite, *P. ramosa*, along with hundreds of other genes of mostly unknown function.

Three LSP haplotypes at this region were observed within one well-studied *D. magna* population (Lake Aegelsee), with strong selection against Haplotype 1 observed during a seasonal *P. ramosa* outbreak. However, an unknown mechanism balances selection against the susceptible haplotype, as demonstrated across a decade of longitudinal field data. A high diversity of additional haplotypes sampled across three continents suggests that this region is a species-wide structural polymorphism. Our results explain previous contradictions regarding the genomic architecture of the *P. ramosa* resistance locus (E locus) but also raise two major questions. First, what is the evolutionary history of these haplotypes, which share little to no detectable sequence homology? Second, what maintains this polymorphism in the long-term, particularly in Lake Aegelsee, where a balancing mechanism prevents susceptible haplotype extinction despite strong selection against it?

### Origins of the large structural polymorphism (LSP) region

The LSP region on chromosome 5 shares characteristics with several types of large-scale polymorphisms known from other organisms. Like supergenes, the chromosome 5 LSP exhibits multiple linked genetic elements, suppressed recombination, discrete phenotypic states, and the maintenance of a balanced polymorphism (Schwander et al. 2014; Thompson and Jiggins 2014; Gutiérrez-Valencia et al. 2021; Berdan et al. 2022). Supergenes are generally associated with well-defined ecologically relevant traits such as sex determination, mating type, or discrete morphotypes. However, LSPs have also been described as regions harboring highly divergent haplotypes with largely non-overlapping gene content and little detectable homology, as observed for hyper-divergent haplotypes in *C. elegans* and related nematodes (Lee et al. 2021; Stevens et al. 2023). Such hyper-divergent haplotypes are not clearly associated with discrete phenotypes and are enriched in immune-related genes. They may instead reflect ancient structural polymorphisms maintained by balancing selection. The LSP region of *D. magna* chromosome 5 also shares several features with these examples—such as extreme haplotype divergence and elevated genetic diversity, but does not fit neatly within either the classical supergene or hyper-divergent haplotype frameworks.

The process which led to the formation of many divergent haplotypes is unclear. Some models point to chromosomal inversion, duplication, deletion, or introgression events (Gutiérrez-Valencia et al. 2021; Villoutreix et al. 2021; Kim et al. 2022). However, the haplotypes at this chromosome 5 region cannot be easily reconstructed as rearrangements of existing genetic elements. Each haplotype contains a substantial number of private genes, with 182 found in the susceptible haplotype and ~90 in each resistant haplotype. In this unusual aspect, the LSP region of chromosome 5 is similar to the previously described *Pasteuria* resistance cluster, which also features numerous divergent haplotypes where homology is difficult to discern (Bento et al. 2017; Naser-Khdour et al. 2024).

Another key feature which distinguishes the LSP region of chromosome 5 from many other known LSPs is the functional implications of its genetic architecture. Supergenes are defined by the interaction of multiple physically linked genetic elements to produce a distinct phenotype. In this context, the genetic architecture of a supergene provides a mechanism to preserve favourable combinations of alleles and minimize potentially unfavourable intermediate or mixed phenotypes (Thompson and Jiggins 2014) (Coadapted gene complexes; Fisher 1930; Dobzhansky 1950). However, the number of elements that comprise the E locus and the way they interact remains unclear. The molecular mechanism of *Pasteuria* resistance is hypothesized to involve the expression and post-translational modification of highly tissue-specific cuticular proteins, which suggests the interaction of multiple genetic elements (Bento et al. 2017). However, our phenotypic data are compatible with a relatively simple genetic model for resistance to *P. ramosa* P20, such as a single gene with two allelic states or a presence/absence polymorphism (Ameline et al. 2021).

The LSP region of chromosome 5 may represent an ancient non-recombining structural polymorphism. This structure may have indeed been more like a supergene in the early stages of its evolution, with two functional haplotypes evolving a mechanism to stop recombination, and thus preserve beneficial combination of allelic variants. Over time, haplotypes may have accumulated mutations (including structural changes) and gradually diverged to such a degree that homology became undetectable (e.g., Meselson effect and the Sheltered Load concept; see Naser-Khdour et al. 2024 for discussion). Given a potentially ancient origin, it would not be unexpected to observe this LSP region to be older than recent speciation events. In this case, haplotypes at the LSP may show a signal of a trans-species polymorphism (TSP) among *Daphnia* as has already been shown for *Pasteuria* resistance genes in three *Daphnia* species (Cornetti et al. 2024).

### Long term persistence of haplotype polymorphism

The *D. magna—P. ramosa* system is well known for its antagonistic coevolution, long-term balancing selection and trans-species polymorphisms (Ameline et al. 2021; Dexter et al. 2023; Cornetti et al. 2024). The association between the LSP region on chromosome 5 and *P. ramosa* resistance suggests that parasite-mediated balancing selection plays a key role in maintaining haplotype diversity. However, the long-term persistence of Haplotype 1 (P20 susceptible) in Lake Aegelsee remains puzzling given the strong selection against it. Ten years of field data show a recurring cycle in which P20 susceptibility declines sharply each summer but reappears at similar frequencies each spring (Fig. 4) (Ameline et al. 2022). This pattern suggests a role for recombination in regenerating susceptible genotypes, as spring hatchlings emerge from sexually produced eggs laid the previous season.

Recombination and segregation shuffle genetic variants within a genome, thus producing new combinations for selection to act upon. While the haplotypes described here do not recombine, they do appear to segregate freely. All possible combinations of the three haplotypes from Lake Aegelsee are viable (Table 1, Supplemental Table 1), and genetic crosses confirm normal Mendelian inheritance (Ameline et al. 2021). Thus, segregation does not produce lethal genotypes that could distort genotype frequencies and explain the persistence of otherwise unfavorable haplotypes. Dominance effects also cannot explain the persistence of P20 susceptibility, as the P20-susceptible allele at the *E* locus is dominant (Ameline et al. 2021). Similarly, masking epistasis from the *C* locus does not fully account for the pattern, as the *cc* genotype (which overrides *E*) is rare in late-season populations (Ameline et al. 2022).

While it remains possible that unknown loci influence selection on the susceptible haplotype, no evidence currently supports this. On the contrary, previous work indicates that segregation patterns at the C and E loci together explain the resistance polymorphism against the P20 isolate extremely well (Ameline et al. 2021; Ameline et al. 2022) and that selection by P20 *Pasteuria* types in the Aegelsee population explains not only the decline in susceptible host genotypes carrying the Haplotype 1, but also the decline of the *cc* host genotype (Fig. 4). One could speculate that some form of selfish genetic element distorts genotype frequencies, as has been shown for balanced polymorphisms in ants (reviewed in Chapuisat 2023), but no evidence for such a mechanism has been found in *D. magna*.

An alternative hypothesis does not rely on recombination or segregation but rather considers fluctuating selection to explain the persistence of P20 susceptible haplotypes. In this scenario, the frequency of P20 resistance declines when P20-like parasite strains are rare due to an innate cost of resistance. *Daphnia* from Lake Aegelsee experience precisely this type of release from *Pasteuria* selection in the spring of each year, as they typically begin to emerge from their resting eggs in early April, about 2 months before *Pasteuria* infection appears in appreciable numbers (Ameline et al. 2022). This cost of resistance could arise from intrinsic fitness differences between alternate alleles at the E locus, or at unrelated loci which co-segregate with the E locus as part of the LSP region. However, this hypothesis is somewhat unsatisfactory in that it would suggest that Haplotype 1 should increase in frequency whenever the parasite is rare, which has not been observed (Fig. 4).

A variation of the fluctuating selection hypothesis concerns a specific cost of P20 resistance, namely an elevated susceptibility to infection by other *P. ramosa* isolates. Previous studies of the *D. magna* – *P. ramosa* system have shown that infection patterns can follow a matching allele model; infection only occurs between specific combinations of host and bacterial genotypes (Luijckx et al. 2013; Bento et al. 2017; Bento et al. 2020; Ameline et al. 2021). Universally resistant or susceptible *D. magna* genotypes do not appear to exist. Therefore, specific combinations of resistance alleles will confer resistance to certain *P. ramosa* genotypes but at the cost of susceptibility to others. This genomic architecture is expected to drive cycles of negative-frequency dependent selection (aka. Red Queen coevolutionary dynamics), as locally adapted *P. ramosa* populations will select against common resistance alleles (Decaestecker et al. 2007; Metzger et al. 2016; Bourgeois et al. 2021; Ebert 2025).

Previous investigation of Lake Aegelsee *Pasteuria* has shown evidence for seasonal succession of *P. ramosa* genotypes, with some genotypes being much more common in earlier months and other genotypes in later months (Ameline et al. 2021). It is therefore possible that Haplotype 1 persists in Lake Aegelsee because the disadvantage of P20 susceptibility is counterbalanced by resistance to one or more early season *P. ramosa* genotypes. Although this hypothesis widely congruent with our understanding of the *D. magna—P. ramosa* system, the fact that we do not see a gradual change in genotype frequencies, but rather an abrupt change associated with hatching from sexual eggs, hints at a more complex process that in some way relates to sexual reproduction.

In conclusion, our study provides a striking example of balancing selection acting on a large structural polymorphism, with pronounced parasite-driven cycles in genotype frequencies. While balancing selection is often inferred from genomic signatures, this system offers a rare phenotypic demonstration of the process in real time. Despite strong and recurrent selection against P20 susceptibility, the susceptible haplotype consistently re-emerges at intermediate frequencies each spring as *Daphnia* hatch from overwintering resting eggs (Ameline et al. 2022). This recurring cycle of selection and persistence underscores the dynamic nature of host-parasite coevolution and highlights the role of balancing selection in maintaining extreme haplotype divergence within natural populations.

Ever since the first models of host-parasite coevolution (Clarke 1976), parasite-driven cycles in allele frequencies have been a key target of empirical studies on coevolutionary mechanisms. Whether the fluctuations observed here align with classic Red Queen dynamics or arise from an alternative, idiosyncratic process remains unclear. The persistence of the susceptible haplotype, despite strong seasonal selection against it, suggests an evolutionary puzzle yet to be fully resolved.

## Supplementary information

HDY-25-A0078-s01

HDY-25-A0078-s02

HDY-25-A0078-s03

HDY-25-A0078-s04

## Data Availability

The raw genomic data generated in this study have been deposited in the NCBI database under BioProject PRJNA1344841. All code necessary to replicate our analyses and graphics can be found at https://github.com/edexter/LSP.
