# Supplementary material for: Rapid evolution of a large structural polymorphism during a bacterial epidemic
Source: Heredity (Edinb). 2025 Nov 24;135(1):1–12. doi: 10.1038/s41437-025-00812-7 (PMC12811391; doi:10.1038/s41437-025-00812-7)
Supplement: Supplementary file 1 — HDY-25-A0078-s01 [file 41437_2025_812_MOESM1_ESM.pdf]

Supplemental tables and figures for Dexter et al. 2025 “Rapid evolution of a large structural polymorphism during a bacterial epidemic”

Supplemental table 1. Observed counts of all possible LSP haplotype combinations for 256 *D. magna* sampled from Lake Aegelsee. Genotypes were assessed from relative mapping depth against the three canonical reference genomes (see main text for details). Cells in grey background are homozygote genotypes, cells with white background are heterozygotes. Note that the data shown in table 1 of the main manuscript excludes samples which were not phenotyped or were homozygous for the recessive C locus allele (which masks the E locus). The data presented here includes all 256 samples.

	Haplotype 1	Haplotype 2	Haplotype 3
Haplotype 1	10	14	18
Haplotype 2	-	48	121
Haplotype 3	-	-	45

Supplemental table 2. Inventory of *Daphnia* genome assemblies used in this manuscript. The resistotypes shown are in reference to *P. ramosa* isolates C1, C19, and P20. Haplotype refers to the LSP-5-1 locus. All clones, except the last entry (which is *Daphnia similis*) are *Daphnia magna*.

Assembly name	Sequencing platform	Resistotype	Origin	Haplotype
CH-H-2015-59	PacBio HiFi	RRS	Switzerland (Aegelsee)	1 Homozygote
CH-H-2015-49	PacBio HiFi	RRS	Switzerland (Aegelsee)	1 Homozygote
CH-H-2014-t2-17-3-4i-13	PacBio HiFi	RRS	Switzerland (Aegelsee)	1 Homozygote
CH-H-2014-t3-12-3-1i-12	PacBio HiFi	RRS	Switzerland (Aegelsee)	1 Homozygote
CH-H-2014-t4-12-3-3	PacBio CLR	SSS	Switzerland (Aegelsee)	1 Homozygote
CH-H-434-inb3-a-1	PacBio CLR	RRR	Switzerland (Aegelsee)	2 Homozygote
FI-SK-58-2-18-4	PacBio CLR	RRR	Finland	2 Homozygote
CH-H-2016-h-34	PacBio CLR	SSS	Switzerland (Aegelsee)	2 Homozygote
CH-H-2299	PacBio CLR	RRR	Switzerland (Aegelsee)	2/3 Heterozygote
DZ-JV-2	PacBio CLR	RRR	Algeria	2 Homozygote
CH-H-t1-10-3-2	PacBio HiFi	RRR	Switzerland (Aegelsee)	3 Homozygote
CN-W1-1	PacBio CLR	RRR	China	3/? Heterozygote
RU-RM1-2	PacBio CLR	RRR	Russia (Moscow)	4 Homozygote
US-SP221-1	PacBio CLR	RRR	USA (Maine)	5 Homozygote
NO-V-7	PacBio CLR	SSS	Norway	6 Homozygote
IL-TY-10	PacBio CLR	SRS	Israel	7 Homozygote
ET-C-1	PacBio CLR	SRR	Ethiopia	8 Homozygote
US-D-3	PacBio CLR	RRR	USA (California)	9 Homozygote
FI-Xinb3	Illumina HiSeq	RSR	Finland	10 Homozygote
NIES clone ASM2063170v1.1 (NCBI accession)	Oxford Nanopore PromethION; Illumina HiSeq	SSS	USA (Minnesota)	11 Homozygote
D.similis_IL-SIM-A20-inb3	PacBio CLR	RRR	Israel	12 Homozygote

Supplemental table 3. Selected gene prediction results for the three LSP haplotypes showing private Fucosyltransferase gene content contained within Haplotype 1 (P20 susceptible). Multiple entries for the same gene represent matches under several different models and databases. Gene prediction was performed with a model trained on RNA-seq and ISO-seq data from the *D. magna* clone FI-Xinb3 using Augustus version 3.4.0. The complete list of functional annotations for private genes for all haplotypes are provided as separate .csv files.

geneID	contig	startPos	stopPos	model	hitName
g1293	3	3237931	3239050	Pfam	Fucosyltransferase, N-terminal
g1293	3	3237931	3239050	Panther	Alpha-1,3-fucosyltransferase
g1326	3	3904619	3905639	Pfam	Glycosyltransferase family 10 (fucosyltransferase) C-term
g1326	3	3904619	3905639	Panther	Alpha-1,3-fucosyltransferase-related

Supplemental figure 1. Whole genome short-reads from 258 previously sequenced *D. magna* individuals from Lake Aegelsee were mapped against each of the reference assemblies. Genotypes at the chromosome 5 LSP region were then assigned to each sample based on the ratio of read depth within the LSP region to the read depth in the adjacent flanking regions on the same chromosome. Points shown in blue indicate homozygous individuals relative to each haplotype, with points shown in red indicating heterozygotes for one copy of the relevant haplotype. Points shown in black indicate samples containing two copies of haplotypes which differ from the reference. Note that from a theoretical perspective, the mapping ratios are expected to be 1.0, 0.5, and 0.0 relative to each haplotype, but a variety of technical aspects (e.g. low-complexity sequence, transposable elements, duplicated genes) cause deviations from this expectation. Nonetheless, the relative mapping depths appear to reliably discriminate each haplotype in an unambiguous manner.

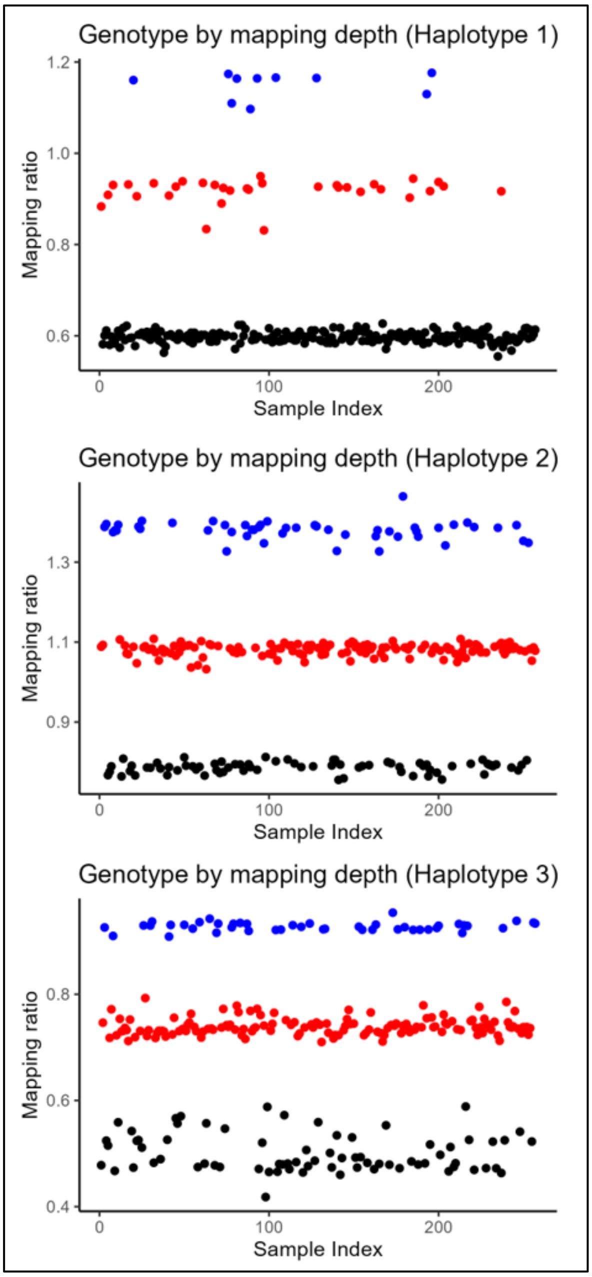

Additional supplemental files

Haplotype1_private.csv: Gene prediction results for haplotype 1 private alleles

Haplotype2_private.csv: Gene prediction results for haplotype 2 private alleles

Haplotype3_private.csv: Gene prediction results for haplotype 3 private alleles
